# Exploration of simultaneous transients between cerebral hemodynamics and the autonomic nervous system using windowed time-lagged cross-correlation matrices: a CENTER-TBI study

**DOI:** 10.1007/s00701-024-06375-6

**Published:** 2024-12-16

**Authors:** Agnieszka Uryga, Cyprian Mataczyński, Adam I. Pelah, Małgorzata Burzyńska, Chiara Robba, Marek Czosnyka, CENTER-TBI high-resolution sub-study participants and investigators, Audny Anke, Ronny Beer, Bo-Michael Bellander, Erta Beqiri, Andras Buki, Manuel Cabeleira, Marco Carbonara, Arturo Chieregato, Giuseppe Citerio, Hans Clusmann, Endre Czeiter, Bart Depreitere, Ari Ercole, Shirin Frisvold, Raimund Helbok, Stefan Jankowski, Daniel Kondziella, Lars-Owe Koskinen, Ana Kowark, David K. Menon, Geert Meyfroidt, Kirsten Moeller, David Nelson, Anna Piippo-Karjalainen, Andreea Radoi, Arminas Ragauskas, Rahul Raj, Jonathan Rhodes, Saulius Rocka, Rolf Rossaint, Juan Sahuquillo, Oliver Sakowitz, Peter Smielewski, Nino Stocchetti, Nina Sundstrom, Riikka Takala, Tomas Tamosuitis, Olli Tenovuo, Andreas Unterberg, Peter Vajkoczy, Alessia Vargiolu, Rimantas Vilcinis, Stefan Wolf, Alexander Younsi, Frederick A. Zeiler

**Affiliations:** 1 Department of Biomedical Engineering, Faculty of Fundamental Problems of Technology, Wroclaw University of Science and Technology, Wroclaw, Poland; 2 Department of Computer Engineering, Faculty of Information and Communication Technology, Wroclaw University of Science and Technology, Wroclaw, Poland; 3 Brain Physics Laboratory, Division of Neurosurgery, Department of Clinical Neurosciences, Addenbrooke Hospital, University of Cambridge, Cambridge, UK; 4 Clinical Department of Anesthesiology and Intensive Care, Faculty of Medicine, Wroclaw Medical University, Wroclaw, Poland; 5 IRCCS Policlinico San Martino, Genoa, Italy; 6 Department of Surgical Sciences and Integrated Diagnostics (DISC), University of Genoa, Viale Benedetto XV 16, Genoa, Italy

**Keywords:** Brain–heart coupling, Traumatic brain injury, Machine learning, Autonomic nervous system

## Abstract

**Background:**

Traumatic brain injury (TBI) can significantly disrupt autonomic nervous system (ANS) regulation, increasing the risk for secondary complications, hemodynamic instability, and adverse outcome. This retrospective study evaluated windowed time-lagged cross-correlation (WTLCC) matrices for describing cerebral hemodynamics–ANS interactions to predict outcome, enabling identifying high-risk patients who may benefit from enhanced monitoring to prevent complications.

**Methods:**

The first experiment aimed to predict short-term outcome using WTLCC-based convolution neural network models on the Wroclaw University Hospital (WUH) database (P_training_ = 31 with 1,079 matrices, P_val_ = 16 with 573 matrices). The second experiment predicted long-term outcome, training on the CENTER-TBI database (P_training_ = 100 with 17,062 matrices) and validating on WUH (P_val_ = 47 with 6,220 matrices). Cerebral hemodynamics was characterized using intracranial pressure (ICP), cerebral perfusion pressure (CPP), pressure reactivity index (PRx), while ANS metrics included low-to-high-frequency heart rate variability (LF/HF) and baroreflex sensitivity (BRS) over 72 h. Short-term outcome at WUH was assessed using the Glasgow Outcome Scale (GOS) at discharge. Long-term outcome was evaluated at 3 months at WUH and 6 months at CENTER-TBI using GOS and GOS-Extended, respectively. The XGBoost model was used to compare performance of WTLCC-based model and averaged neuromonitoring parameters, adjusted for age, Glasgow Coma Scale, major extracranial injury, and pupil reactivity in outcome prediction.

**Results:**

For short-term outcome prediction, the best-performing WTLCC-based model used ICP-LF/HF matrices. It had an area under the curve (AUC) of 0.80, vs. 0.71 for averages of ANS and cerebral hemodynamics metrics, adjusted for clinical metadata. For long-term outcome prediction, the best-score WTLCC-based model used ICP-LF/HF matrices. It had an AUC of 0.63, vs. 0.66 for adjusted neuromonitoring parameters.

**Conclusions:**

Among all neuromonitoring parameters, ICP and LF/HF signals were the most effective in generating the WTLCC matrices. WTLCC-based model outperformed adjusted neuromonitoring parameters in short-term but had moderate utility in long-term outcome prediction.

**Supplementary Information:**

The online version contains supplementary material available at 10.1007/s00701-024-06375-6.

## Introduction

Traumatic brain injury (TBI) ranks among the leading causes of death and disability worldwide. The Lancet Neurology Commission on TBI, published in 2017 [49] and then in 2022 [50], reported that TBI was estimated to constitute one of the top three causes of injury-related death and disability, with 50 million–60 million people sustaining a TBI each year, worldwide. Despite advancements in intensive care, little progress has been made in reducing TBI-related morbidity and mortality [50]. Modern neurocritical care, driven by neural networks and advanced biosignal processing, has the potential to revolutionize personalized diagnostic and treatment approaches [30, 47]. Brain trauma as an acute biomechanical event is characterized by multiple pathophysiological processes that develop over time and are not limited to the brain [61].

Under normal conditions, the brain regulates cerebral blood flow (CBF) through cerebral autoregulation, a process that is partially controlled by the autonomic nervous system (ANS) [13, 40, 59]. Conversely, the brain influences cardiac function through the sympathetic and parasympathetic branches of the ANS, which consists of multisynaptic pathways from myocardial cells back to peripheral ganglionic neurons and further to central preganglionic and premotor neurons [67]. This bidirectional communication creates an intricate network, allowing the brain and heart to continuously exchange information [35]. However, this interdependence can be significantly modified after acute brain injury, which might contribute to cerebral hypoperfusion and secondary injury after TBI [19, 42, 63, 73]. Understanding and following therapeutic modulation of brain–heart interactions may be an option for improving outcome [35, 75].

Shortly after TBI, there is an increase in sympathetic activity and catecholamine levels. The initial state of hyperactivity of the sympathetic branch resulted in organ vasoconstriction and decreased perfusion [21]. This high sympathetic tone persists after TBI, with circulating catecholamine levels remaining high for up to 10 days, which is a potentially protective mechanism, designed to maintain cerebral perfusion in the presence of increased intracranial pressure (ICP), but it also has several adverse effects [36]. Although the most important time frame for ANS dysfunction analysis has not yet been clearly defined, recent studies have suggested that the first 72 hours post-injury may be crucial [8, 27, 78, 79]. In some patients, paroxysmal sympathetic hyperactivity (PSH) syndrome develops and is characterized by episodic tachycardia, hypertension, tachypnea, hyperpyrexia, diaphoresis, and abnormal motor posturing. The general prevalence of PSH is up to one-third of patients with moderate and severe TBI, however, because PSH is often misinterpreted in clinical practice, the appropriate incidence of PSH is likely greater [58].

Several advanced signal-processing methods have been used to describe brain–heart interactions. Analytic techniques such as recurrence plots, cross-correlation functions, and wavelet analysis have been applied to identify transient elevations in heart rate (HR) and ICP in the TBI cohort [25]. Principal dynamic mode analysis has shown that adding HR into a two-input model of cerebral hemodynamics (with arterial blood pressure and CO_2_ concentration as inputs) significantly reduces prediction error [52]. To explore phase‒causal links between brain and heart oscillations, cross-frequency coupling functions have been proposed [68]. Additionally, a complex network approach based on visibility graphs has been introduced to analyze network topological measures for detecting brain–heart communication within the system [24].

The computational complexity of these advanced methodologies has limited their adoption in clinical practice [26]. Conversely, in traditional analyses, high-resolution signals are averaged into a single value, which eliminates short-term patterns. Transient episodes can be captured by correlating time series at the individual level, rather than at the cohort level. An alternative method, that may overcome these limitations is windowed time-lagged cross-correlation (WTLCC).

The WTLCC characterizes the fine-grained dynamics between time series. Unlike a cross-correlation computed over the entire series, which may provide a limited view of the dynamics between neuroparameters, WTLCC uses short sliding windows [5]. When applied to high-resolution monitoring, it generates hundreds of correlation matrices (heatmaps) for a patient and thousands of them for a cohort.

This retrospective study aimed to investigate the utility of WTLCC matrices in describing brain–heart interactions after brain trauma. We hypothesize that quantifying these dynamics during the acute phase after brain injury may enable the identification of high-risk patients who could benefit from enhanced monitoring to prevent complications and improve outcome prediction after TBI.

## Materials and methods

### Study design

**Fig. 1:**
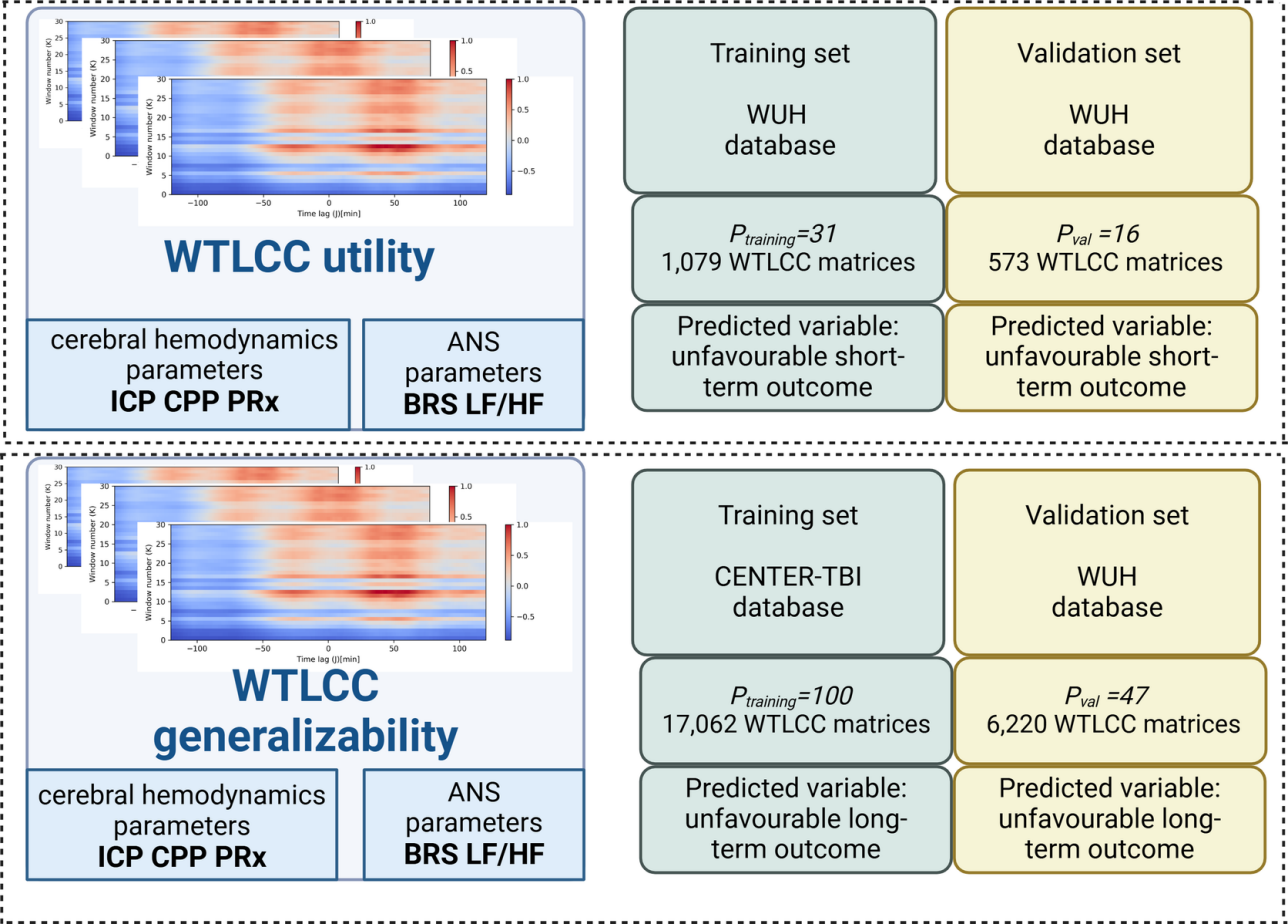
Study design. The aim of the first experiment (‘WTLCC utility’) was to investigate to the utility of windowed time-lag cross-correlation (WTLCC) matrices, which describe cerebral hemodynamics-autonomic nervous system (ANS) interactions for predicting short-term outcome. For cerebral hemodynamics parameters, intracranial pressure (ICP), cerebral perfusion pressure (CPP), pressure reactivity index (PRx) were used, and baroreflex sensitivity (BRS) and low-to-high component ratio of heart rate variability (LF/HF) were used as ANS metrics. This experiment was performed exclusively on the Wroclaw University Hospital (WUH) database. The second experiment (‘WTLCC generalizability’) aimed to evaluate the utility of WTLCC matrices for predicting long-term outcome in a larger, external database of TBI patients. For this purpose, the CENTER-TBI database was used for training, and the WUH database was used for validation

The first experiment (‘WTLCC utility’) investigated the effectiveness of the WTLCC-based model for predicting short-term outcome in TBI patients, assessing whether it differentiates short-term outcome better than averaged neuromonitoring parameters. This experiment was conducted exclusively on the Wroclaw University Hospital (WUH) database. The second experiment (‘WTLCC generalizability’) assessed the model’s utility for predicting long-term outcome in a broader, external TBI dataset. For this purpose, the CENTER-TBI database was used for training and the WUH database was used for validation. We also evaluated whether the WTLCC model offered improved differentiation for long-term outcomes over averaged neuromonitoring parameters. The detailed study design is shown in Fig. 1.

### Ethical approval

Ethical approval for the retrospective analysis of the WUH database was obtained from the Bioethics Committee at WUH, Poland, under approval KB-133/2023. The CENTER-TBI study (European Commission grant 602,150) was conducted in accordance with all relevant laws of the European Union that are directly applicable or of direct effects and all relevant laws of the country where the recruiting sites were located, including but not limited to, the relevant privacy and data protection laws and regulations (the “Privacy Law”), the relevant laws and regulations on the use of human materials, and all relevant guidance relating to clinical studies from time to time in force, including but not limited to, the ICH Harmonized Tripartite Guideline for Good Clinical Practice (CPMP/ICH/135/95) (“ICH GCP”) and the World Medical Association Declaration of Helsinki entitled “Ethical Principles for Medical Research Involving Human Subjects.” Informed consent by the patients and/or the legal representative/next of kin was obtained, according to the local legislations, for all patients recruited in the Core Dataset of CENTER-TBI and documented in the e-CRF. Ethical approval was obtained for each recruiting site from the appropriate local ethics committee, and the full list of approvals is available on the website: https://www.center-tbi.eu/project/ethical-approval. This analysis adheres to the Guidelines for Strengthening the Reporting of Observational Studies in Epidemiology (STROBE) Statement (Supplementary materials).

### Study population

The WUH database includes patients admitted to the intensive care unit (ICU) of the WUH from 2014–2019. All patients were diagnosed with acute brain injury (TBI or aneurysmal subarachnoid haemorrhage (aSAH)). In this study, the following inclusion criteria were used: patients aged 18 years or older, with available outcome at hospital discharge and long-term outcome, ICP sensor implementation, and hemodynamic stability at the start of monitoring, with good quality signals during the first three days of monitoring. None of the patients underwent craniectomy. A flow chart is presented in Supplementary Fig. 1. All patients were treated according to guidelines applicable at the time of admission (for aSAH [20]and for TBI [12]). The study group was homogenous in terms of the severity of the injury and treatment protocol. In the TBI cohort, all patients suffered predominantly from severe TBI (severe = Glasgow Coma Scale (GCS) score of 8 or less). In patients who required craniectomy, monitoring ended after surgery. In the aSAH cohort the decision concerning treatment with surgical clipping or endovascular coiling of the aneurysm, was based on the patient’s condition and physician’s interest and was performed within 24 h after admission to the hospital. In the ICU, all patients were classified according to the GCS score, with the majority of patients assessed as being in severe condition. Angiography with computer tomography (angio-CT) was used to localize the ruptured aneurysm. The Hunt and Hess (H–H) scale and the World Federation of Neurological Surgeons (WFNS) grading scale were used to classify aSAH. The extent of haemorrhage was evaluated with the Fisher scale.

The CENTER-TBI is a large multicenter European project that aims to better understand and improve the care of patients with TBI. Patients were recruited prospectively between the beginning of 2015 and the end of 2017 from 21 medical centers across Europe. All patients were treated following current evidence-based guidelines for TBI [15]. Detailed information on the data collection is available on the study website (https://www.center-tbi.eu/data/dictionary). Version CENTER Core 3.0 of the CENTER-TBI dataset was used in this study. Among the 2138 patients in the ICU included in the CENTER-TBI data collection, a subgroup of 282 patients, named the high-resolution CENTER-TBI substudy, had high-frequency digital signals from ICU monitoring. In this study, we applied the following inclusion criteria: patients over 16 years of age, with available hospital discharge status and follow-up data after six months, ICP sensor implantation, no craniotomy performed, and good-quality signals recorded during the first three days of monitoring. The flow chart for patient inclusion is presented in Supplementary Fig. 2. All patients were treated according to guidelines applicable at the time of admission and the group was homogenous in terms of the treatment protocol.

### Signal monitoring and processing

**Fig. 2:**
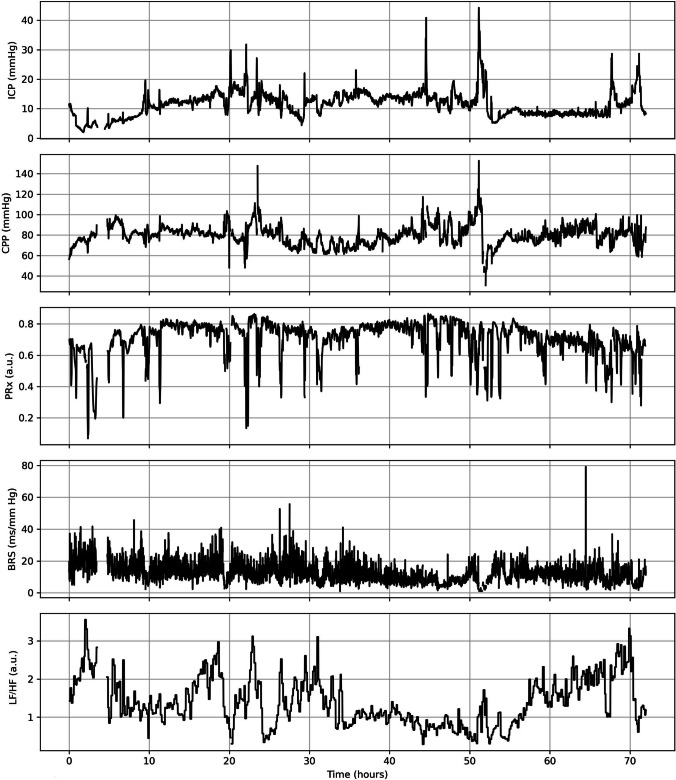
An examplary time series of cerebral hemodynamics parameters: intracranial pressure (ICP), cerebral perfusion pressure (CPP), and pressure reactivity index (PRx), as well as autonomic nervous system parameters: baroreflex sensitivity (BRS) and the ratio between the low (LF, 0.04–0.15 Hz) and high (HF, 0.15–0.40 Hz) frequency ranges of heart rate variability (LF/HF) during the first three days (72 h) of recordings in traumatic brain injury patient hospitalized at Wroclaw University Hospital (WUH)

In the WUH database, the ICP was measured invasively using an intraparenchymal probe (Codman MicroSensor ICP Transducer, Codman & Shurtleff, Randolph, MA, USA) inserted into the frontal cortex. Arterial blood pressure (ABP) was measured invasively in the radial or femoral artery using a pressure transducer (Argon Standalone DTX Plus™, Argon Medical Devices Inc. Plano, TX, USA). The signal was recorded with a sampling frequency of 200 Hz using ICM + software (Cambridge Enterprise Ltd., Cambridge, UK). In the CENTER-TBI database, the ICP was measured via intraparenchymal strain gauge probe (Codman ICP MicroSensor, Codman & Shurtlef Inc., Raynham, MA, USA) or parenchymal fiber optic pressure sensor (Camino ICP Monitor, Integra Life Sciences, Plainsboro, NJ, USA). ABP was measured via a radial or femoral arterial line connected to a pressure transducer (Baxter Healthcare Corp., CardioVascular Group). The signal was recorded with a sampling frequency of 100 Hz or higher using ICM + software and/or the Moberg CNS Monitor (Moberg Research Inc., Ambler, PA, USA). In both databases, multimodal signal recording was performed within the first 24 h after onset. In this study, we used the first three days of neuromonitoring (Fig. 2). The mean values of all the signals and derived parameters were calculated using waveform time integration over 60-s intervals; therefore, the discrepancy in the sampling frequency could be neglected.

### Outcome

In the WUH database, outcome was assessed in the short-term (hospital discharge) and long-term (after three months) using Glasgow Outcome Scale (GOS), which was dichotomized as favourable (4–5) or unfavourable (1–3). The assessment was performed by one experienced neurointesivist (M.B.) and was adjusted for assessment of independence when assessment was performed during discharge, as recommended [77]. In the CENTER-TBI database, long-term outcome (after six months) was assessed using the Glasgow Outcome Scale Extended (GOS-E). The outcome scores were dichotomized as favourable (lower than moderate disability or better [GOS-E score of 5–8]) or unfavourable (upper severe disability or worse [GOS-E score of ≤ 4]) on the basis of the dichotomization utilized in the other trials [3].

### Cerebral hemodynamics parameters

Cerebral perfusion pressure (CPP) was defined as the difference between the mean ABP and ICP. Cerebral autoregulation was assessed using pressure reactivity index (PRx), calculated as the Pearson linear correlation coefficient between slow waves in the ABP and the ICP signal. First, the signals were averaged over 10 s intervals to isolate the slow changes, and then the correlation coefficient was assessed in 5 min moving average windows updated for 10 s [22].

### Autonomic nervous system metrics

Baroreflex sensitivity (BRS) was assessed in the time domain using the sequential cross-correlation method proposed by Westerhof et al. [76]. It was calculated as the slope of the regression line between 10 s segments of the systolic peak-to-peak interval and the corresponding systolic pressure time series derived from the ABP signal. Heart rate variability was assessed in the frequency method using the Lomb–Scargle periodogram as the ratio between the low range (LF, 0.04–0.15 Hz) and high range (HF, 0.15–0.40 Hz), termed LF/HF [51].

### Windowed time-lagged cross-correlation

**Fig. 3:**
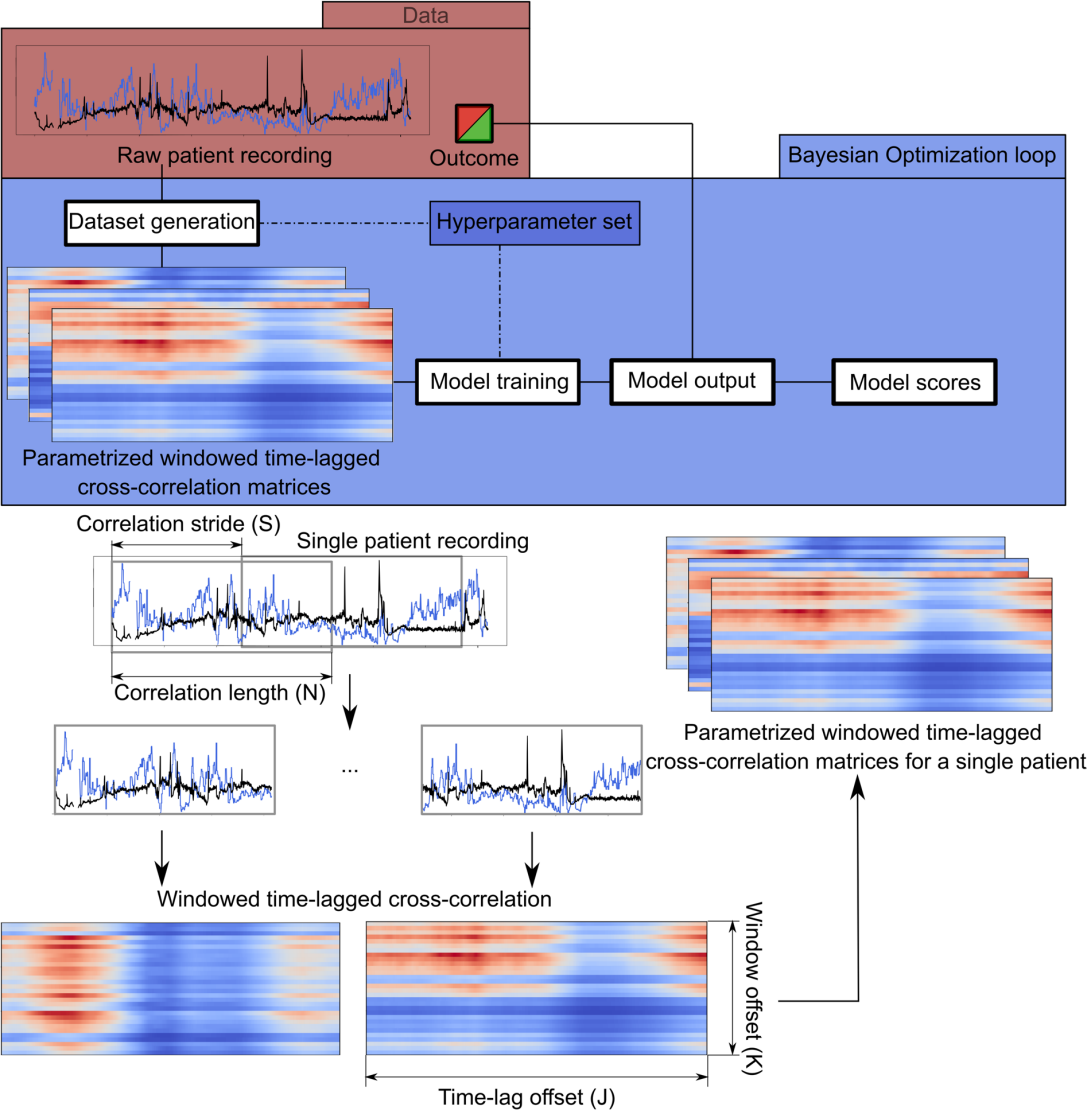
The pipeline of the analysis uses windowed time-lagged cross-correlation (WTLCC) *(Upper panel)* WTLCC is applied to two series *I*_*1*_*(n)* and *I*_*2*_*(n)* of length *N,* creating a matrix with *K* rows and *J* columns. *(Lower panel)* A custom convolutional neural network (CNN) was used as a general approximator for the task of finding a mapping from the WTLCC matrices to a binary variable of unfavourable/favourable outcome. The details of WTLCC and CNN are presented in the Supplementary materials

The WTLCC analysis is illustrated in Fig. 3. Correlated parameters (*I*_*1*_ and *I*_*2*_) included the first 72 h of cerebral hemodynamics parameters (ICP, CPP, and PRx) and ANS metrics (LF/HF and BRS). WTLCC applied to two series *I*_*1*_*(n)* and *I*_*2*_*(n)* of length* N* creates a matrix with *K* rows and *J* columns, where each row *k* corresponds to a time-lagged cross-correlation for the *k*^*th*^ window between both signals [10]. Each value in this row is a time-lagged correlation value of the windowed signals with the lag *l*_*k,j*_ for the *j*^*th*^ column being equal to *l*_*k,j*_ = *j-J/2* samples. Next, a window of length *N* is sampled from the signal by moving it beginning with a stride value *S*, thus creating multiple overlapping windows from a single patient (Supplementary Fig. 3). The hyperparameters used for heatmap generation were adjustable from the following sets: *N*: 360 min, 720 min, 1080 min; *S*: 15 min, 30 min, 60 min, 90 min; and *K*: 15, 30, 60. By default, the time lag (*J*) was set as a constant 240 min (with a 1-min resolution, moving 120 min both way). All the hyperparameters of the WTLCC matrices were optimized using Bayesian optimization technique. The details of the optimization procedure are presented in the Supplementary Data.

### Convolutional neural network

A custom convolutional neural network (CNN) was used as a general approximator for finding a mapping from the WTLCC matrices to a binary variable of unfavourable/favourable outcome. The exact structure, training details and hyperparameters of the networks can be found in Supplementary Table 1. A total of 2016 models were trained. We score the models using aggregate metrics computed for all the matrices from a single patient. The scores are concatenated using a simple heuristic that computes the means of all the scores assigned to every matrix from a single patient and checks whether the score exceeds the threshold of 0.5 (see the analysis pipeline in Fig. 3). The learning procedure is described in detail in the Supplementary Data. The performance of the CNN model was assessed using a receiver operating characteristic (ROC) curve with the area under the curve (AUC), accuracy and F1 score.

### XGBoost model

To compare the performance of the features learned from the WTLCC-based model and clinical metadata, which are commonly used for outcome prediction, we propose an extension to the experiments. We generate a simple tabular classifier using the gradient-boosted trees algorithm (XGBoost) [16] to predict short and long-term outcome on the basis of one of the two potential feature sets: (1) average values of neuroparameters (ICP, CPP, PRx, BRS, LF/HF) adjusted for clinical features from the established Corticoid Randomization after Significant Head Injury (CRASH) model (age, Glasgow Coma Scale (GCS), pupil reactivity, major extracranial injury) [57]; (2) WTLCC-based model embeddings adjusted for clinical features from the CRASH model. The model embeddings are feature vectors generated by applying the feature extraction component of the best-performing CNN model (identified during hyperparameter optimization) to the WTLCC matrix. The data split used for training this classifier is the same as that used for training the model, reducing information leakage between the training and validation datasets. In the XGBoost model for long-term outcome prediction, where the CENTER-TBI database was used as the training set, the P_training_ was 92, because of missing data about GCS (*n* = 5) and pupil reactivity (*n* = 3).

### Statistical analysis

The normality of the data was assessed using the Shapiro‒Wilk test. Because the normality condition was not met for most of the analyzed parameters, nonparametric tests were applied. The differences in median values were tested using the Mann–Whitney *U* test. For categorical data, the Pearson CHI^2^ test (Fisher exact test) was used. The level of significance was set at 0.05. Data are presented as the median (first–third quartile) unless indicated otherwise. Statistical analysis was performed using STATISTICA 13 (Tibco, Palo Alto, CA, USA).

## Results

### Study population

The WUH database included 47 patients with acute brain injury, consisting of 38 with TBI and 9 with aSAH. The clinical characteristics of the WUH cohort are presented in Table 1. The inclusion and exclusion criteria are detailed in the flow chart (Supplementary Fig. 1). The median age was 38 (28–63) years, with 15 (32%) females. Patients were in moderate to severe condition, with a GCS score of 7 (5–8). In the aSAH group the haemorrhage was classified as severe, with mFisher score of 4 (3–4), WFNS score of 5 (3–5), and H–H scale score of 5 (4–5). Unfavourable short-term outcome was found in 35 (74%) patients in the group. At the 3-month follow-up, 21 (45%) of the patients had unfavourable long-term outcome. The CENTER-TBI database includes 100 patients with TBI. The clinical characteristics of this cohort are presented in Table 1. The detailed inclusion and exclusion criteria are presented in the flow chart (Supplementary Fig. 2). The median age was 53 (36–64) years, with 25 (25%) being female. Patients were in moderate to severe condition, with a GCS score of 7 (3–11). Within this group, at the 6-month follow-up, 51 (51%) of the patients had unfavourable long-term outcome.

**Table 1:** Baseline clinical characteristics of patients from the Wroclaw University Hospital (WUH) database and the CENTER-TBI database. Data are presented as median (lower quartile-upper quartile) or number (percentage)

Characteristics of total group	WUH (*N* = 47)	CENTER-TBI (*N* = 100)
TBI	38 (81%)	100 (100%)
aSAH	9 (19%)	0
Age [years]	38 (28–63)	53 (36–64)
Female	15 (32%)	25 (25%)
Glasgow Coma Scale	7 (5–8)	7 (3–11)^a^
ISS	36 (26–50)	32 (25–41)
Major extracranial injuries	25 (53%)	39 (39%)
^b^Cause of injury:		
Road traffic incident	20 (53%)	40 (40%)
Incident fall	14 (37%)	34 (34%)
Other nonintentional injuries	2 (4%)	4 (4%)
Violence/Assault	1 (3%)	12 (12%)
Suicide attempt	0	0
Other/Unknown	1 (3%)	10 (10%)
^b^Pupillary reactivity:		
Bilaterally reacting	29 (76%)	77 (77%)
Unilaterally reacting	7 (18%)	8 (8%)
Bilaterally nonreacting	2 (6%)	12 (12%)
NA	0	3 (3%)
^c^CT characteristics:		
Contusion	24 (51%)	59 (59%)
Epidural hematoma	4 (9%)	20 (20%)
Cerebral Hematoma	15 (32%)	32 (32%)
Traumatic SAH	16 (34%)	77 (77%)
^d^Outcome:		
Unfavourable short-term outcome,* n(%)*	35 (74%)	NA
Unfavourable long-term outcome,* n(%)*	21 (45%)	51 (51%)

*TBI*, traumatic brain injury; *aSAH*, aneurysmal subarachnoid haemorhhage; *ISS*, injury severity scale; *CT*, computed tomography; *NA*, data were not available; ^a^ data concerning the Glasgow Coma Scale were not available for *n* = 5 patients; ^b,c^ variables available for TBI patients, where for ^c^ more than one is possible; ^d^ In the WUH database short-term outcome was assessed at hospital discharge and long-term outcome was assessed after 3 months using Glasgow Outcome Scale (GOS); in the CENTER-TBI, long-term outcome was assessed after 6 months using the Glasgow Outcome Scale Extended (GOS-E)

## Experiment 1: WTLCC utility

### Selected hyperparameters for WTLCC matrices

The WUH database was randomly divided into two separate groups for training and validation (*P*_*training*_ = *31, P*_*val*_ = *16*). Across all the experiments (see details in the ‘Convolutional neural network’ section), the following hyperparameters received the best score metrics: parameters I_1_ – ICP, and I_2_ – LF/HF. The WTLCC matrices parameters were as follows: length (*N*) of 1080 min, correlation step (*S*) of 90 min, and a number of windows (*K*) of 60.

### WTLCC-based model for short-term outcome

**Fig. 4:**
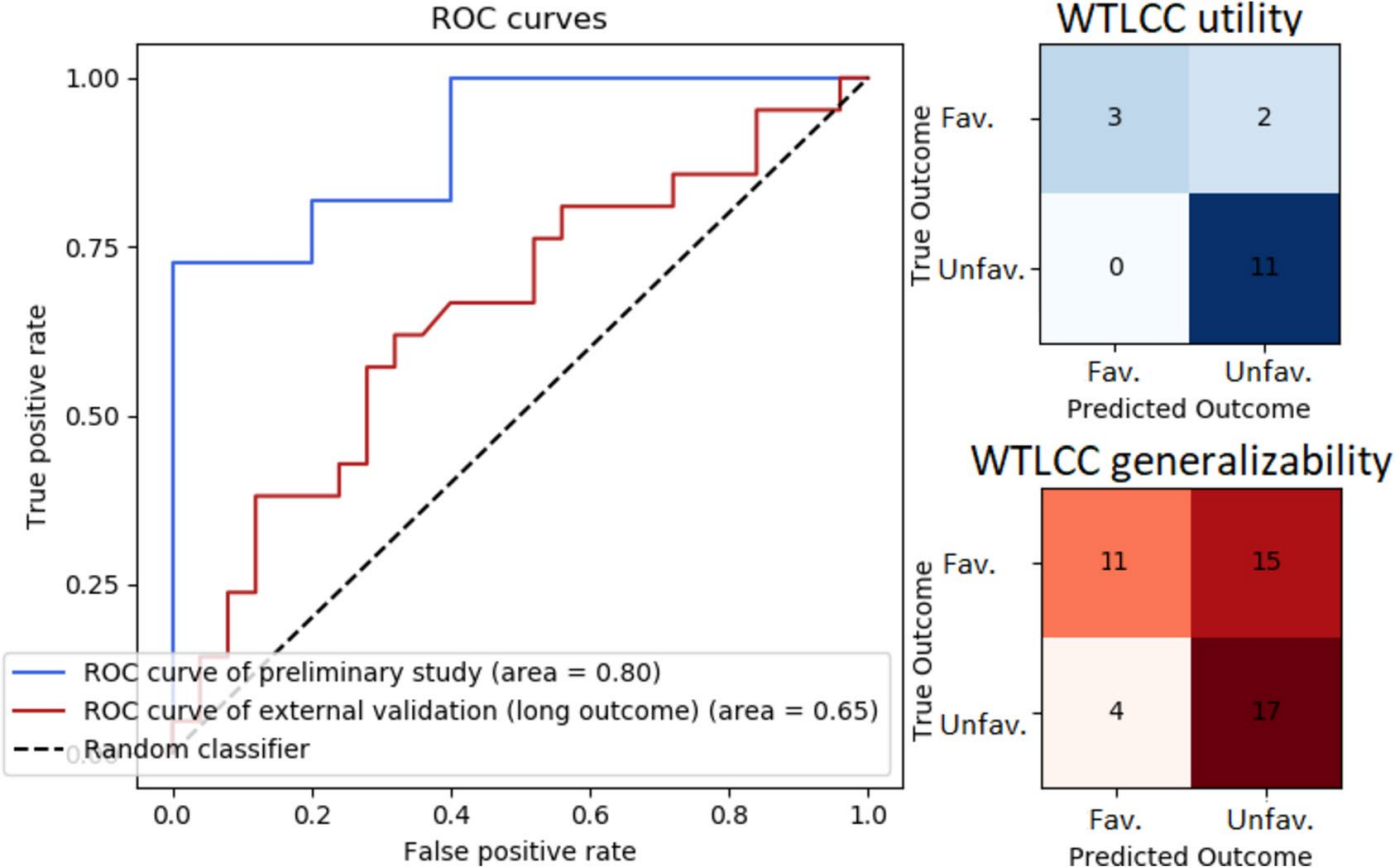
A receiver operating characteristic (ROC) curve and confusion matrix for the convolutional neural network (CNN) model utilizing windowed time-lagged cross-correlation (WTLCC) in two experiments. The first experiment (‘WTLCC utility’) aimed to predict short-term outcome and was trained and validated on the Wroclaw University Hospital (WUH) database (*P*_*training*_ = *31* with 1,079 matrices, *P*_*val*_ = *16* with 573 matrices). The second experiment (‘WTLCC generalizability’) aimed to predict long-term outcome and was trained on the CENTER-TBI database and validated on WUH (*P*_*training*_ = 100 with 17,062 matrices*, P*_*val*_ = 47 with 6,220 matrices). Abbreviations: Fav. – Favourable outcome; Unfav.– Unfavourable outcome

The CNN model was trained on 1,079 matrices and validated on 573 matrices. The best-performing CNN, used to map acute-phase WTLCC matrices to short-term outcome, achieved an accuracy of 88%, with an F1 score of 0.92 and an AUC of 0.80. The ROC curve and confusion matrix are presented in Fig. 4.

### Averages of neuromonitoring parameters vs. short-term outcome

A comparison of the average neuromonitoring parameters by short-term outcome in the WUH database is presented in Table 2. Notably, none of the neuromonitoring parameters averaged over the first three days were significant predictors of unfavourable outcome.

**Table 2:** Cerebral hemodynamic parameters and autonomic nervous system metrics for patients with favourable and unfavourable short-term and long-term outcome. Data are presented as median (lower quartile-upper quartile)

*Database: WUH (n* = *47)*			
Parameter	Favourable short-term outcome *n* = 12	Unfavourable short-term outcome *n* = 35	*p*-value
ICP [mm Hg]	12.9 (10.2–14.2)	11.8 (9.1–14.9)	0.854
CPP [mm Hg]	74.3 (71.5–78.3)	72.6 (65.0–77.4)	0.311
PRx [a.u.]	0.60 (0.46–0.67)	0.63 (0.55–0.71)	0.360
BRS [ms/mm Hg]	6.4 (4.2–9.2)	6.8 (4.5–8.7)	0.931
LF/HF [a.u.]	0.79 (0.49–1.15)	0.85 (0.33–1.55)	0.970
*Database WUH (n* = *47)*			
Parameter	Favourable long-term outcome *n* = 26	Unfavourable long-term outcome *n* = 21	*p*-value
ICP [mm Hg]	12 (10–15)	13 (9–15)	0.957
CPP [mm Hg]	73 (69–77)	74 (65–77)	0.940
PRx [a.u.]	0.57 (0.46–0.61)	0.69 (0.62–0.74)	< 0.001
BRS [ms/mm Hg]	6.7 (4.8–9.6)	5.6 (4.0–8.1)	0.226
LF/HF [a.u.]	0.96 (0.54–1.56)	0.59 (0.29–1.36)	0.167
*Database CENTER-TBI (n* = *100)*			
Parameter	Favourable long-term outcome *n* = 49	Unfavourable long-term outcome *n* = 51	*p*-value
ICP [mm Hg]	12 (9–15)	13 (10–16)	0.488
CPP [mm Hg]	73 (67–78)	67 (62–74)	0.011
PRx [a.u.]	0.48 (0.32–0.60)	0.56 (0.40–0.67)	0.048
BRS [ms/mm Hg]	8.1 (4.5–11.3)	6.3 (4.5–13.1)	0.901
LF/HF [a.u.]	1.76 (0.90–2.65)	1.01 (0.69–1.65)	0.002

*WUH*, Wroclaw University Hospital database; *ICP*, intracranial pressure; *CPP*, cerebral perfusion pressure; *PRx*, pressure reactivity index; *BRS*, baroreflex sensitivity; *LF/HF*, ratio between the low (LF, 0.04–0.15 Hz) and high (HF, 0.15–0.40 Hz) frequency ranges of heart rate variability

### WTLCC-based model vs. neuromonitoring parameters adjusted for CRASH features in short-term outcome prediction

We compared WTLCC-based model embeddings with average values of cerebral hemodynamics and ANS metrics (ICP, CPP, PRx, BRS, LF/HF), after adjustment for clinical features from the established CRASH model (age, GCS, pupil reactivity, major extracranial injury), in the short-term outcome prediction task. This predictive modelling was performed using the XGBoost algorithm (see details in the ‘Methods’ section). The adjusted neuromonitoring parameters had an accuracy of 75%, with an F1 score of 0.82 and an AUC of 0.71. The adjusted WTLCC-based model embeddings had an accuracy of 88%, with an F1 score of 0.92 and an AUC of 0.80.

## Experiment 2: WTLCC generalizability

### Selected hyperparameters for WTLCC matrices

The training process in experiment 2 was performed independently of experiment 1, and both databases (CENTER-TBI and WUH) were not mixed in any stage of analysis. The CENTER-TBI database was used as the training set (*P*_*training*_ = *100)* and the WUH database was used as the validation set (*P*_*val*_ = *47)*. The following hyperparameters received the best score metrics across all the experiments: parameters I_1_ – ICP, and I_2_ – LF/HF. The WTLCC matrix parameters were as follows: length (*N*) of 1080 min, correlation step (*S*) of 15 min, and a number of windows (*K*) of 60.

### WTLCC-based model for long-term outcome

The CNN model was trained on 17,062 matrices and validated on 6,220 matrices. The best-score custom CNN, used for the task of finding a mapping from the acute-phase WTLCC matrices to long-term outcome, had an accuracy of 59%, with an F1 score of 0.64 and an AUC of 0.65. The ROC curve and confusion matrix are presented in Fig. 4.

### Averages of neuromonitoring parameters vs. long-term outcome

A comparison of neuromonitoring parameters between groups with favourable and unfavourable long-term outcome is presented in Table 2. In the WUH database, PRx was higher in patients with unfavourable outcome than in those with favourable (0.69 ± 0.12 *vs.* 0.57 ± 015; *p* < 0.001).

In the CENTER-TBI database, CPP [mm Hg] (67 ± 11 vs. 73 ± 12, *p* = 0.011) and LF/HF (1.01 ± 0.96 *vs*. 1.76 ± 1.75, *p* = 0.002) were lower, whereas the PRx was higher (0.56 ± 0.27 *vs*. 0.48 ± 0.27, *p* = 0.048) in patients with unfavourable outcome than in those with favourable.

### WTLCC-based model vs. neuromonitoring parameters adjusted for CRASH features in long-term outcome prediction

We compared WTLCC-based model embeddings with average values of cerebral hemodynamics and ANS metrics, after adjustment for clinical features from the CRASH model, in the long-term outcome prediction task. This predictive modelling was performed using the XGBoost algorithm (see details in the ‘Methods’ section). The adjusted neuromonitoring parameters had an accuracy of 65%, with an F1 score of 0.67 and an AUC of 0.66. The adjusted WTLCC-based model embeddings had an accuracy of 63%, with an F1 score of 0.62 and an AUC of 0.63.

## Discussion

In this study, we propose a new method to explore the simultaneous transients between cerebral hemodynamics and ANS by employing an artificial intelligence-based model utilizing acute-phase WTLCC matrices. Among all the neuromonitoring parameters, ICP and LF/HF signals were the most effective in generating the WTLCC matrices. For short-term outcome prediction, the WTLCC-based model performed better than average values of the neuromonitoring parameters after adjusting for clinical features used in the CRASH model. However, in long-term outcome prediction, the utility of the WTLCC-based model was moderate and comparable with the mean values of the neuroparameters.

The WTLCC has been previously applied to study the relationship between simultaneous time series describing human perceptions and performance [64]. To our knowledge, this is the first study to explore the utility of WTLCC in predicting patient outcome after acute brain injury. The proposed solution preserves the temporal dynamics between neuromonitoring parameters. By segmenting data into time windows, WTLCC generates hundreds of correlation matrices per patient, similar to a ‘snapshot’, each capturing the relationship between cerebral hemodynamics and ANS parameters while accounting for potential delays and time-related changes. This approach transforms a one-dimensional time series into two-dimensional matrices, which can be analyzed similarly to images using a CNN model.

The incidence of dysautonomia or paroxysmal sympathetic hyperactivity (PSH) is approximately 8% to 33% of TBI patients [32, 58]; however its incidence may be difficult to diagnose because these patients typically have significant neurologic and systemic injuries. In severe TBI hyperactivity of the sympathetic nervous system as an adaptive response to damaged tissues can result in damage to the myocardium and other critical organs [45]. Most research suggests that brain injury is commonly associated with increases in sympathetic activity, which can alter the regulatory function of critical organs such as the heart via hemodynamic changes [54]. It is also likely that brain injury alters the fine balance between the sympathetic and parasympathetic arms of the autonomic nervous system, resulting in an imbalance of the homeostatic mechanisms that maintain normal organ system function and their interactions with each other. Therefore, investigating of the mechanisms of autonomic dysfunction can guide advancements in monitoring and treatment paradigms to improve the acute survival and long-term prognosis of TBI patients [46].

In the task of finding a mapping from the WTLCC matrices to a binary variable of unfavourable/favourable outcome, ICP and LF/HF were identified as the most accurate time series. ICP is a complex modality that should not be reduced to its mean value, as it reflects cerebral compensatory mechanisms and indirectly regulates cerebral blood flow [23]. Recent studies have reported alterations in ANS activity in response to changes in ICP [33, 62]. However, the ICP-ANS relationship is not straightforward and rather nonlinear [14, 65]. The LF/HF ratio is often used as an indicator of sympathovagal balance. Although it should not be interpreted as a ‘zero-sum’ system [66], low LF/HF, indicating high parasympathetic activity with the withdrawal of the sympathetic branch, has been associated with poor outcome and increased mortality rates [18, 39, 55]. Increased ICP, which is commonly observed after TBI, causes increased sympathetic activity, which in turn results in hypertension, increased heart rate, and catecholamine hypersecretion. Catecholamines increase the contractility of the heart despite increased vascular resistance [6]. On the other hand, previous studies have shown that the parasympathetic system might be triggered by an increase in ICP, leading to vasodilatation to preserve cerebral blood flow, which in turn produces an increase in arterial brain blood volume and consequently a further increase in ICP [31]. It has been demonstrated that in patients with poor outcome, BRS remains low during an increase in ICP, whereas in patients with good outcome, BRS increases with higher ICP [56, 11, 55].

ANS metrics, including heart rate variability (HRV) parameters, are influenced by age, sex, functional capacity, and chronic comorbidities, and measuring them in critically ill patients is accompanied by potential difficulties [69]. Studies where basal autonomic function can be captured, including elective surgery [1, 43] may provide powerful mechanistic insights since autonomic changes can be individualized and referenced to pre-insult normal levels [44]. Despite possible problems and limitations, HRV analysis has been performed in intensive care for the last three decades. It has been shown that brain-injured patients have reduced HRV, whereas recovery of HRV is associated with improved outcome [38]. In a study of the effects of sepsis, age, sedation, catecholamines, and illness severity on sympathovagal balance (LF/HF), an LF/HF ratio < 1.5 was shown to be associated with sepsis and mortality [48]. Moreover, a negative correlation between LF/HF and the SOFA score has been reported [4]. Sykora et al. [71] reported that a decreased LH/HF ratio in a TBI cohort of patients was significantly associated with increased mortality, independent of ICP and CPP. Bodenes et al. [9] reported that lower LF/HF, and Shannon entropy values at admission were associated with higher ICU mortality. Moreover, HRV measured (including LF/HF) on admission enables the prediction of outcome in the ICU or on day 28, independent of the admission diagnosis, treatment, and mechanical ventilation requirements.

The prediction of outcome after brain injury is still a challenge. Two of the most recognized and reported models of outcome prediction after TBI are CRASH [57] and the International Mission for Prognosis and Analysis of Clinical Trials (IMPACT) [70]. However, both models have limitations. They are based on demographic and clinical data related to the primary injury. According to current research extending prognostic models with early monitoring data of physiological signals may improve the accuracy of outcome prediction [7, 37, 60]. Furthermore, the CRASH or IMPACT models are designed for long-term outcome prediction but lack the ability to guide life-or-death decisions for individual patients. According to the study of Eagle et al., these models incorrectly predicted that nearly 1 in 5 patients would have an unfavourable outcome or die [28]. In our study, we showed that in the prediction of short-term outcome the WTLCC-based model performed better than cerebral hemodynamics and ANS metrics, adjusted for clinical characteristics included in the CRASH model (age, GCS score, pupil reactivity, major extracranial injury). However, in terms of their ability to predict long-term outcome, their performance was comparable. While short-term outcome may be affected not only by the severity of brain injury but also by the extent of systemic disorders, the function of peripheral organs, and transient changes in cerebral hemodynamics and ANS activity [41, 72], long-term recovery after brain injury may also depend on care pathways and rehabilitation [11, 53]. Current studies have shown that patients with TBI who are treated in an ICU are a highly heterogeneous group. Therefore clustering by glucose variations and brain biomarkers (such as glial fibrillary acidic protein, S100 calcium-binding protein B and others), could be the best clinical descriptors of disease trajectories in the ICU. However, the implementation of brain biomarkers in clinical practice has not yet been widely accomplished [2]. New metrics of the brain–heart interactions can serve as ‘biomarkers’ of healthy brain–heart interaction, making their assessment valuable in understanding the pathological mechanisms of TBI.

Guideline-based care for moderate to severe TBI patients relies on various pharmacological agents as treatment cornerstones. Sedatives are used to manage ICP and suppress cerebral metabolic demand, whereas vasopressor agents are utilized to maintain CPP targets [17]. HRV metrics are sensitive to sedation levels [56]. Propofol has been found to significantly reduce sympathetic nerve activity and diminish reflex increases in sympathetic nerve activity; however, this observation was based on a relatively small group (n = 10) of healthy volunteers aged 21–37 years [29]. Conversely, a recent study conducted by Froese et al. on 475 patients hospitalised with brain trauma demonstrated that infusions of commonly administered sedatives and vasopressor agents do not impact cerebrovascular reactivity [34]. Therefore, the effects of sedatives and vasopressors on the ANS and cerebral hemodynamics are still under investigation, something which is not accounted for in our study.

The proposed pipeline aims to explore the relationships between the ANS and cerebral hemodynamics metrics. It takes advantage of neural networks as general approximators. With fixed network hyperparameters, we constrained the Bayesian optimization algorithm to prioritize extracting the most information from the WTLCC matrices rather than fully optimizing the model structure for the best possible scores. This modification in dataset creation required adjustments in the training process, prompting us to include both training and WTLCC hyperparameters in the optimization process. However, this approach has limitations. First, assessing model performance with limited data is challenging, as a small patient sample affects score resolution and requires an external testing dataset. To address this, we used two completely independent databases in the second experiment, showing that even in this scenario, the model achieved moderate results in terms of outcome prediction. Additionally, we restricted the hyperparameter options to a predefined set to ensure that the assumptions were met.

## Limitations

This study has several limitations. In the first experiment, we used the WUH database exclusively for training and validation. Given the limited data, we were unable to use a separate test set in this preliminary study. For the second experiment, we address this limitation by using two separate databases, CENTER-TBI as the training dataset and WUH as the validation dataset, to assess the generalizability of our findings. Although we acknowledge that this approach has some limitations, it allows us to minimise overfitting concerns and assess model performance across independent datasets. Additionally, as a short-term outcome metric we used GOS assessed at hospital discharge in the WUH database. However, this assessment was performed by experienced specialist and the questionnaire was adaptive to the aspects of independence if necessary and according to recommendations [77]. The impairment in ANS regulation observed in TBI patients outweighs the transient changes inherent in and inseparable from the critical care environment (changes in body temperature and position, respiratory rate, tidal volume, nursing manoeuvres, alterations in the medical therapy and drugs), as reported in previous studies [71]. However, we cannot be certain that they do not influence ANS parameters, therefore this should be considered the primary limitation of this study. Given the heterogeneity between traumatic brain injury (TBI; *n* = 38 (81%)) and aneurysmal subarachnoid haemorrhage (aSAH; *n* = 9; 19%)) in the WUH cohort of patients, we recognize the potential challenges in combining these subgroups, especially in examining cerebral hemodynamics.

## Conclusions

Among all the neuromonitoring parameters, ICP and LF/HF were the most effective in generating the WTLCC matrices. For short-term outcome prediction, the WTLCC-based model performed better than average values of the neuromonitoring parameters after adjusting for clinical features used in the CRASH model. However, in long-term outcome prediction, the utility of the WTLCC-based model was moderate and comparable with the mean values of the neuroparameters. Further research with a larger TBI patient cohort is needed to confirm these findings.

## Supplementary Information

Below is the link to the electronic supplementary material.

Supplementary file1 (DOCX 215 KB)

## Data Availability

The data that support the findings of this study belong to the CENTER-TBI project (https://www.center-tbi.eu/), but restrictions apply to the availability of these data, which were used under license for the current study (Approval No. 514) and are not publicly available. Access to the data can be obtained upon approval from the CENTER-TBI project committee. Source codes for the analysis are available at https://github.com/AUTOMATIC-BRAIN-ANS/WTLCC-Transients-NN. The dataset generated and/or analyzed during the current study belonging to Wrocław Medical University Hospital (Poland) is not publicly available, but a reasonable request to the corresponding author will be considered.
