# Supplementary material for: Exploration of simultaneous transients between cerebral hemodynamics and the autonomic nervous system using windowed time-lagged cross-correlation matrices: a CENTER-TBI study
Source: Acta Neurochir (Wien). 2024 Dec 16;166(1):504. doi: 10.1007/s00701-024-06375-6 (PMC11649841; doi:10.1007/s00701-024-06375-6)
Supplement: Supplementary file 1 — Supplementary file1 (DOCX 215 KB) [file 701_2024_6375_MOESM1_ESM.docx]

**Supplementary Data**

**Materials and methods**

**Optimization of WTLCC parameters**

The training length was chosen uniformly from integers between 20 and 100, while the learning rate was uniformly sampled between 0.005 and 0.01. The optimization target was to maximize the area under the receiver operating curve (ROC-AUC) classification score. Bayesian optimization models were used as the scoring function, based on selected hyperparameters, to search for the maximum classification score by exploring different hyperparameter sets.

**Learning procedure and network structure**

The hyperparameters of the networks are shown in Supplementary Table 1. Across all the experiments, a total of 2016 models were trained on two separate machines—one with Intel i7-11700, 16 core CPU and Nvidia RTX 3080 Ti with 12 GB of VRAM and the second with Ryzen 9 3900XT 12 core CPU and Nvidia RTX 3090 with 24 GB of VRAM. All of the models were trained via balanced binary cross-entropy loss, with the balancing factor being positive to negative class balance. Both the initial learning rate and the number of epochs were optimized from model to model. Early stopping with a patience of 15 epochs was used to stop poorly performing models from continuing. The Adam optimizer (Kingma et. Al, 2014) was used along with the gradient accumulation method, which accumulated gradients from 16 batches of 256 examples before performing an optimizer step. The gradients were not clipped during training. Model performance in the validation dataset was logged at the end of every epoch, and running averages of the training dataset were recorded every 10 steps. The source code used for analysis is available at <https://github.com/AUTOMATIC-BRAIN-ANS/WTLCC-Transients-NN>.

**References**

Kingma, D. and Ba, J.L. (2014) Adam: A Method for Stochastic Optimization. Computer Science, 1-15. <https://arxiv.org/abs/1412.6980>

**Supplementary Table 1.**

Feature Extractor Structure.

| **Feature Extractor Structure:** | |
| --- | --- |
| 1 x Convolution block #1 | |
| Conv2d | 32 filters, 7x7, 1 stride |
| BatchNorm2d | 32 norms |
| ReLu activation | -- |
| 1 x Convolution block #2 | |
| Conv2d | 64 filters, 5x5, 1 stride |
| BatchNorm2d | 64 norms |
| ReLu activation | -- |
| 3 x Residual Block – residual connection from input to last activation | |
| Conv2d | 64 filters, 3x3, 1 stride |
| BatchNorm2d | 64 norms |
| ReLu activation | -- |
| Conv2d | 64 filters, 3x3, 1 stride |
| BatchNorm2d | 64 norms |
| Residual sum | BatchNorm output + layer input |
| ReLU | -- |
| Adaptive Pooling (1 feature from each channel) | |
| **Classifier Structure:** | |
| Fully connected Layer + ReLU | 128 neurons, 0.3 dropout probability, ReLU activation |
| Fully connected Layer + ReLU | 128 neurons, 0.3 dropout probability, ReLU activation |
| Fully connected Layer | 1 neuron |
| Sigmoid | |

**
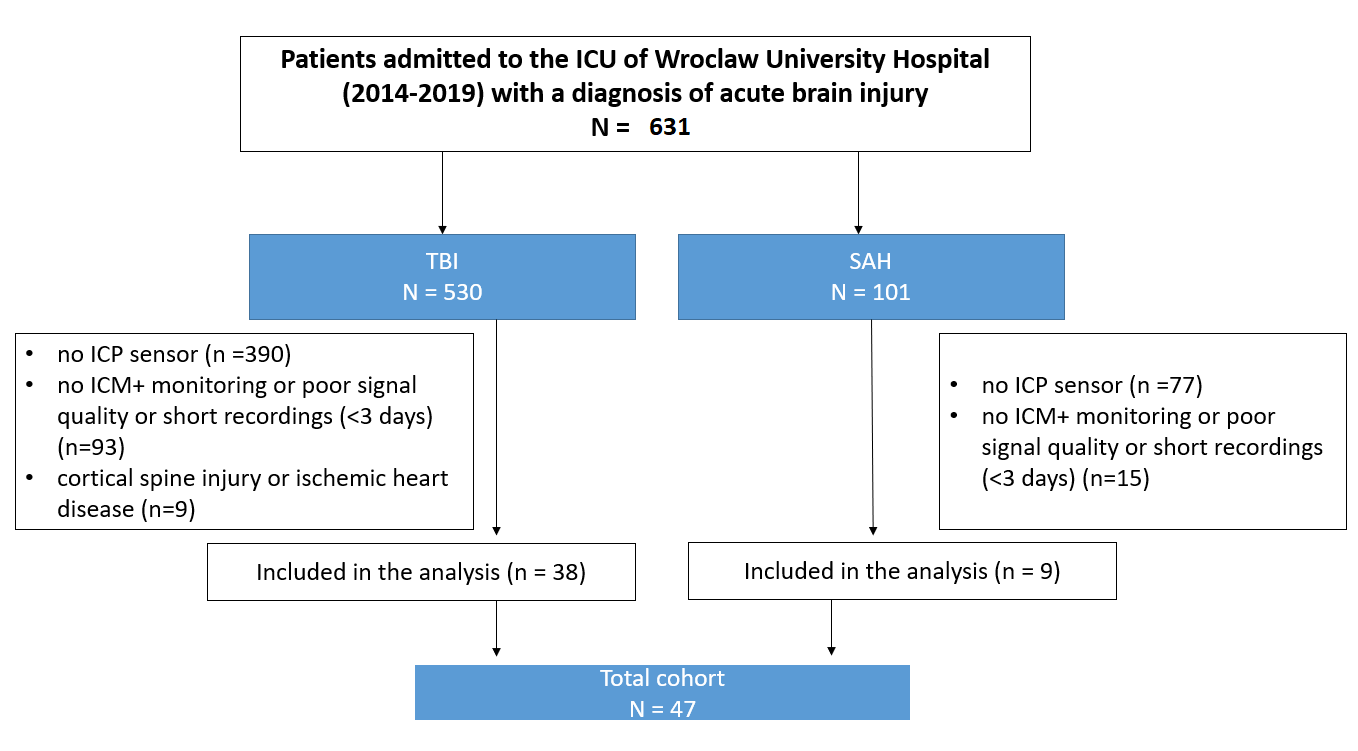
**

**Supplementary Figure 1**

Flow chart of the study design based on recordings from Wroclaw University Hospital. Abbreviations: ICP, intracranial pressure; ICU, intensive care unit; ICM+, intensive care monitoring software

**
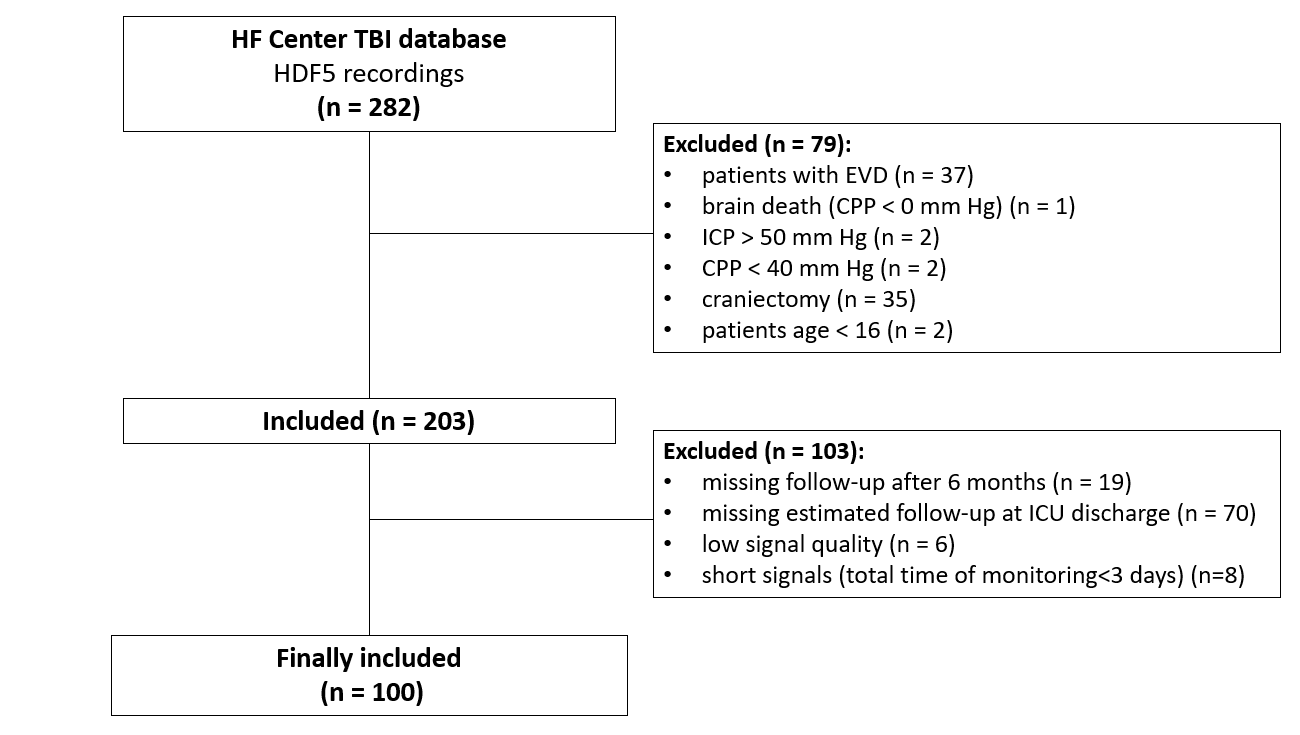
**

**Supplementary Figure 2**

Flow chart of the study design based on recordings from the CENTER-TBI database. Abbreviations: EVD – external ventricular drainage, CPP – cerebral perfusion pressure, ICP – intracranial pressure, ICU – intensive care unit


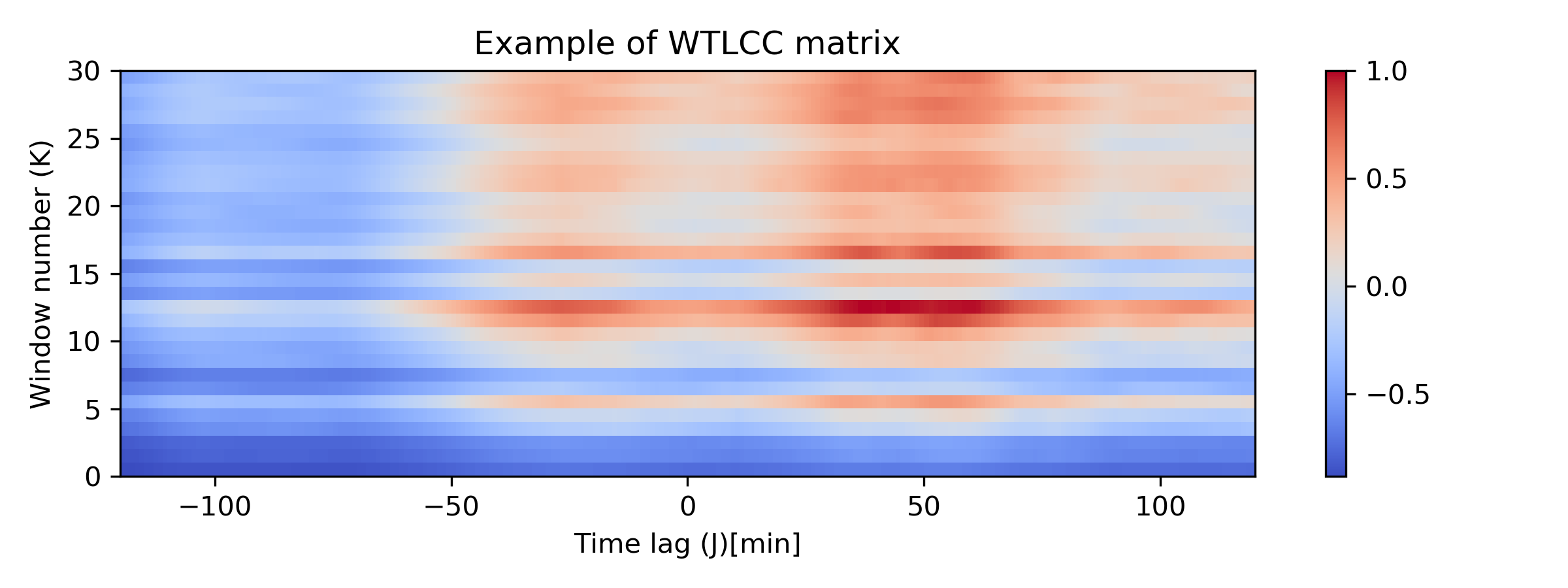


**Supplementary Figure 3**

An exemplary representation of windowed time-lagged cross-correlation (WTLCC) matrix. WTLCC is applied to two series *I_1_(n)* and *I_2_(n)* of length *N,* creating a matrix with *K* rows and *J* columns, where each row *k* corresponds to a time-lagged cross-correlation for the *k^th^* window between both signals. Each value in this row is a time-lagged correlation value of the windowed signals, with the lag *l_k,j_* for the *j^th^* column being equal to *l_k,j_* = *j-J/2* samples (a negative number of samples means that the window of series *I_1_* is translated such that the number of samples forward in relation to the series *I_2_*).

“Strengthening the Reporting of Observational Studies in Epidemiology (STROBE)” statement guidelines.

|  | Item No. | Recommendation | Page  No. |
| --- | --- | --- | --- |
| **Title and abstract** | 1 | (*a*) Indicate the study’s design with a commonly used term in the title or the abstract | 1 |
|  |  | (*b*) Provide in the abstract an informative and balanced summary of what was done and what was found | 4 |
| Introduction | | | |
| Background/rationale | 2 | Explain the scientific background and rationale for the investigation being reported | 5,6 |
| Objectives | 3 | State specific objectives, including any prespecified hypotheses | 6 |
| Methods | | | |
| Study design | 4 | Present key elements of study design early in the paper | 6 |
| Setting | 5 | Describe the setting, locations, and relevant dates, including periods of recruitment, exposure, follow-up, and data collection | 6-8 |
| Participants | 6 | (*a*) *Cohort study*—Give the eligibility criteria, and the sources and methods of selection of participants. Describe methods of follow-up  *Case‒control study*—Give the eligibility criteria, and the sources and methods of case ascertainment and control selection. Give the rationale for the choice of cases and controls  *Cross-sectional study*—Give the eligibility criteria, and the sources and methods of selection of participants | 6-8 |
|  |  | (*b*) *Cohort study*—For matched studies, give matching criteria and number of exposed and unexposed  *Case‒control study*—For matched studies, give matching criteria and the number of controls per case |  |
| Variables | 7 | Clearly, define all outcomes, exposures, predictors, potential confounders, and effect modifiers. Give diagnostic criteria, if applicable | 7-8 |
| Data sources/measurement | 8* | For each variable of interest, give sources of data and details of methods of assessment (measurement). Describe comparability of assessment methods if there is more than one group | 8 |
| Bias | 9 | Describe any efforts to address potential sources of bias | NA |
| Study size | 10 | Explain how the study size was arrived at | 7-8 |

| Quantitative variables | 11 | Explain how quantitative variables were handled in the analyses. If applicable, describe which groupings were chosen and why | 7-9 |
| --- | --- | --- | --- |
| Statistical methods | 12 | (*a*) Describe all statistical methods, including those used to control for confounding | 9 |
|  |  | (*b*) Describe any methods used to examine subgroups and interactions |  |
|  |  | (*c*) Explain how missing data were addressed |  |
|  |  | (*d*) *Cohort study*—If applicable, explain how loss to follow-up was addressed  *Case‒control study*—If applicable, explain how matching of cases and controls was addressed  *Cross-sectional study*—If applicable, describe analytical methods taking account of sampling strategy |  |
|  |  | (*e*) Describe any sensitivity analyses |  |
| Participants | 13* | (a) Report numbers of individuals at each stage of study—e.g. numbers potentially eligible, examined for eligibility, confirmed eligible, included in the study, completing follow-up, and analyzed | 11 |
|  |  | (b) Give reasons for nonparticipation at each stage |  |
|  |  | (c) Consider use of a flow diagram |  |
| Descriptive data | 14* | (a) Give characteristics of study participants (e.g. demographic, clinical, social) and information on exposures and potential confounders | 11-12 |
|  |  | (b) Indicate number of participants with missing data for each variable of interest |  |
|  |  | (c) *Cohort study*—Summarize follow-up time (e.g., average and total amount) |  |
| Outcome data | 15* | *Cohort study*—Report numbers of outcome events or summary measures over time | 11-12 |
|  |  | *Case‒control study—*Report numbers in each exposure category, or summary measures of exposure |  |
|  |  | *Cross-sectional study—*Report numbers of outcome events or summary measures |  |
| Main results | 16 | (*a*) Give unadjusted estimates and, if applicable, confounder-adjusted estimates and their precision (e.g., 95% confidence interval). Make clear which confounders were adjusted for and why they were included | 11-13 |
|  |  | (*b*) Report category boundaries when continuous variables were categorized |  |
|  |  | (*c*) If relevant, consider translating estimates of relative risk into absolute risk for a meaningful time period |  |

| Other analyses | 17 | Report other analyses done—e.g. analyses of subgroups and interactions, and sensitivity analyses | NA |
| --- | --- | --- | --- |
| Key results | 18 | Summarize key results with reference to study objectives | 13 |
| Limitations | 19 | Discuss limitations of the study, taking into account sources of potential bias or imprecision. Discuss both direction and magnitude of any potential bias | 15 |
| Interpretation | 20 | Give a cautious overall interpretation of results considering objectives, limitations, multiplicity of analyses, results from similar studies, and other relevant evidence | 13-16 |
| Generalisability | 21 | Discuss the generalisability (external validity) of the study results | 16 |
| Other information | |  |  |
| Funding | 22 | Give the source of funding and the role of the funders for the present study and, if applicable, for the original study on which the present article is based | 2-3 |

*Give information separately for cases and controls in case‒control studies and, if applicable, for exposed and unexposed groups in cohort and cross-sectional studies.

**Note:** An Explanation and Elaboration article discusses each checklist item and gives the methodological background and published examples of transparent reporting. The STROBE checklist is best used in conjunction with this article (freely available on the Web sites of PLoS Medicine at http://www.plosmedicine.org/, Annals of Internal Medicine at http://www.annals.org/, and Epidemiology at http://www.epidem.com/). Information on the STROBE Initiative is available at [www.strobe-statement.org](http://www.strobe-statement.org)
